# Crystal structures reveal phosphorylation-dependent disruption of the heat shock protein 70-CHIP interface: A compensatory G132N variant restores binding affinity

**DOI:** 10.1016/j.cstres.2026.100166

**Published:** 2026-03-13

**Authors:** Mariah Stewart, Chathura Paththamperuma, Colleen McCann, Kelsey Cottingim, Huaqun Zhang, Rian DelVecchio, Ivy Peng, Erica Fennimore, Jay C. Nix, Morcos N. Saeed, Kathleen George, Katherine Makaroff, Meagan Colie, Ethan Paulakonis, Michael F. Almeida, Adeleye J. Afolayan, Nicholas G. Brown, Richard C. Page, Jonathan C. Schisler

**Affiliations:** 1 The McAllister Heart Institute, The University of North Carolina at Chapel Hill, Chapel Hill, NC 27599, USA; 2 Department of Pharmacology, The University of North Carolina at Chapel Hill, Chapel Hill, NC 27599, USA; 3 Department of Chemistry and Biochemistry, Miami University, Oxford, OH 45056, USA; 4 Molecular Biology Consortium, Beamline 4.2.2, Advanced Light Source, Lawrence Berkeley National Laboratory, Berkeley, CA 94720, USA; 5 Department of Pediatrics, Children’s Research Institute and Cardiovascular Research Center, Medical College of Wisconsin, Milwaukee, WI 53226, USA; 6 Department of Pathology and Lab Medicine, and Computational Medicine Program, The University of North Carolina at Chapel Hill, Chapel Hill, NC 27599, USA

**Keywords:** HSP70, Protein quality control, CHIP (STUB1), Post-translational modifications, Co-chaperone

## Abstract

Heat shock protein 70 (HSP70) and its E3 ligase co-chaperone CHIP (*STUB1*) form a critical quality-control complex that directs client proteins toward folding or degradation. Phosphorylation of HSP70 at a conserved threonine in the C-terminal tail influences the fate of clients during cellular stress, yet the structural basis for this regulation remains unclear. Here, we present crystal structures of the CHIP tetratricopeptide repeat (TPR) domain bound to unphosphorylated and phosphorylated HSP70 C-terminal peptides at 1.6-1.9 Å resolution. Phosphate occupancy at Thr636 (HSPA1A numbering) causes steric clashes and electrostatic repulsion within the TPR-binding groove, decreasing affinity by more than 10-fold, as shown by biolayer interferometry and fluorescence polarization. Molecular dynamics simulations confirm destabilization of key hydrogen bonds. A structure-guided G132N substitution in CHIP introduces new hydrogen bonds to the phosphate group, restoring affinity for phosphorylated peptides in isolated TPR domains without losing native ubiquitination activity. However, in full-length CHIP, interface modifications do not restore phosphorylation-impaired stable binding but yield only partial recovery of transient interactions in cells, indicating additional context-dependent constraints on HSP70-CHIP regulation. These findings reveal the atomic mechanism by which phosphorylation impairs HSP70-CHIP interaction during stress and demonstrate that targeted interface engineering can compensate for post-translational changes in isolated domains. Overall, the results explain how cells switch chaperone-mediated triage pathways and offer a framework for understanding how proteostasis becomes dysregulated in neurodegenerative diseases and cancer.

## Highlights

• Crystal structures reveal how Thr636 phosphorylation disrupts HSP70–CHIP binding.
• Phosphate clashes eliminate two key hydrogen bonds at the TPR interface.
• Engineered G132N CHIP restores affinity for phosphorylated HSP70 in isolated TPR.
• Targeted interface engineering overrides phosphorylation control of chaperone triage.

## Graphical Abstract

**Figure:**
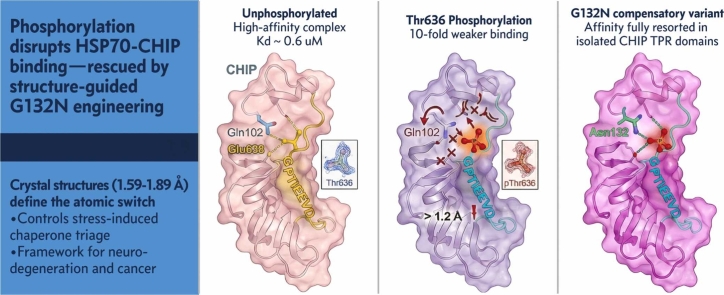

## Introduction

**Fig. 1:**
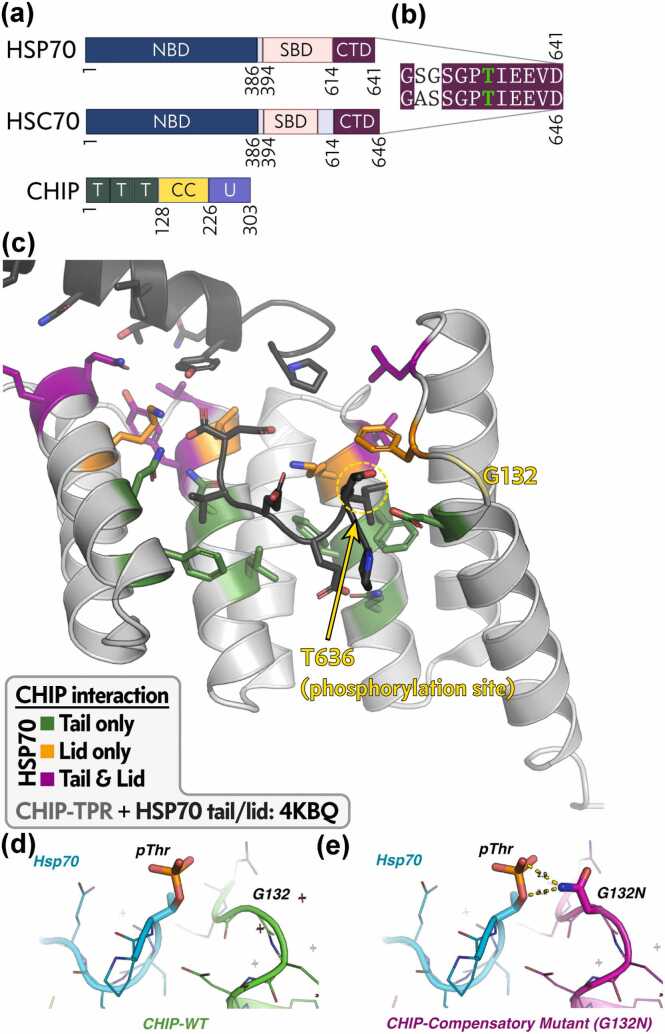
Domain organization and in silico rationale for compensatory CHIP variant. (a) Schematics of HSP70, HSC70, and CHIP depicting key domains: nucleotide-binding domain (NBD), substrate-binding domain (SBD), C-terminal domain (CTD) with EEVD tail, tetratricopeptide repeat (TPR), coiled-coil (CC), and U-box (U). (b) Sequence alignment of the C-terminal tails of human HSP70 (HSPA1A) and HSC70 (HSPA8), highlighting the conserved GPTIEEVD motif and phosphorylation site (T636 in HSP70; green). (c) Previously solved structure of CHIP-TPR in complex with HSP70 helical lid + tail domain (PDB 4KBQ). CHIP-TPR in light gray; HSP70 lid + tail in dark gray. CHIP-TPR residues interacting with tail only (forest green), lid only (magenta), or both (orange). (d) In silico model of wild-type CHIP-TPR interacting with phosphorylated threonine (pThr-636) of HSP70 tail, showing disrupted interactions. (e) In silico model of G132N CHIP-TPR interacting with pThr-636 HSP70 tail, with yellow dashed lines indicating predicted new hydrogen bonds from the asparagine side chain.

Cellular protein quality control integrates the heat shock response with ubiquitin-proteasome and autophagy-lysosome pathways to maintain proteostasis under basal conditions and during stress. Heat shock protein 70 (HSP70/*HSPA1A*) and its constitutive homolog HSC70 (*HSPA8*) are central ATP-dependent chaperones that recognize exposed hydrophobic segments on non-native clients, promoting refolding or triage to degradation machineries.^1^, ^2^, ^3^, ^4^, ^5^ Client fate is dictated by co-chaperones that bind the conserved C-terminal EEVD motif (Figure 1a). The E3 ubiquitin ligase CHIP (*STUB1*) binds this motif via its tetratricopeptide repeat (TPR) domain, inhibiting HSP70 ATPase activity and directing clients to proteasomal degradation^6^, ^7^, ^8^ (Figure 1a). In contrast, HOP (*STI1*) binding favors refolding by enhancing nucleotide exchange.^9^

Post-translational modifications of HSP70 provide rapid control of co-chaperone selection. Phosphorylation at Thr636 (HSPA1A numbering) within the GPTIEEVD sequence (Figure 1b), induced by kinases during oxidative or proteotoxic stress, markedly reduces CHIP affinity while preserving or enhancing HOP binding.^10^, ^11^ This switch redirects clients from degradation toward refolding or alternative pathways, a mechanism implicated in acute stress adaptation and chronic proteostasis failure in neurodegeneration and cancer.^12^, ^13^ Although biochemical studies established phosphorylation sensitivity, the atomic basis for disrupted CHIP binding remained undefined, and strategies to bypass this regulation were untested. Dysregulation of this phosphorylation-dependent switch alters client triage, contributing to proteotoxic stress accumulation in neurodegeneration and enhanced survival signaling in cancer.^12^, ^13^, ^14^ For example, impaired CHIP-mediated degradation of tau promotes its aggregation in Alzheimer’s disease models.^15^, ^16^

Here, we report high-resolution crystal structures of CHIP-TPR bound to unphosphorylated and phosphorylated HSP70 C-terminal peptides. These reveal phosphate-induced steric and electrostatic clashes that destabilize the interface. Molecular dynamics simulations confirm loss of key hydrogen bonds. Structure-guided engineering of CHIP residue Gly132 to asparagine introduces compensatory hydrogen bonds to the phosphate, restoring affinity in isolated TPR domains. Biophysical assays with full-length proteins do not rescue, indicating additional constraints. These findings define the molecular biasing controlling stress-dependent chaperone triage and demonstrate proof-of-concept interface engineering to override post-translational regulation.

## Results

### In silico rationale for compensatory engineering

Previous structures of CHIP-TPR bound to an extended HSP70 helical lid + tail segment (PDB 4KBQ) revealed bipartite contacts: some CHIP residues interact only with the EEVD tail, others only with the lid, and a subset with both (Figure 1c).^8^ Modeling phosphate onto Thr636 within this interface predicted steric clashes and loss of hydrogen bonds from the Thr hydroxyl to CHIP Gln102 and Glu638 (Figure 1d). Gly132, located adjacent to the predicted phosphate position, appeared ideally placed to accommodate an asparagine side chain capable of forming new hydrogen bonds to the phosphate oxygens (Figure 1e). We therefore generated the G132N variant for testing.

### Crystal structures of CHIP-TPR bound to unphosphorylated and phosphorylated HSP70 tail peptides

We determined structures of human CHIP-TPR (residues 21-154) in complex with HSP70 C-terminal octapeptides in unphosphorylated (GPTIEEVD) and phosphorylated (GP(pT)IEEVD) forms at 1.89 Å (PDB 9DYA) and 1.59 Å (PDB 9DYB) resolution, respectively (Table 2).

**Fig. 2:**
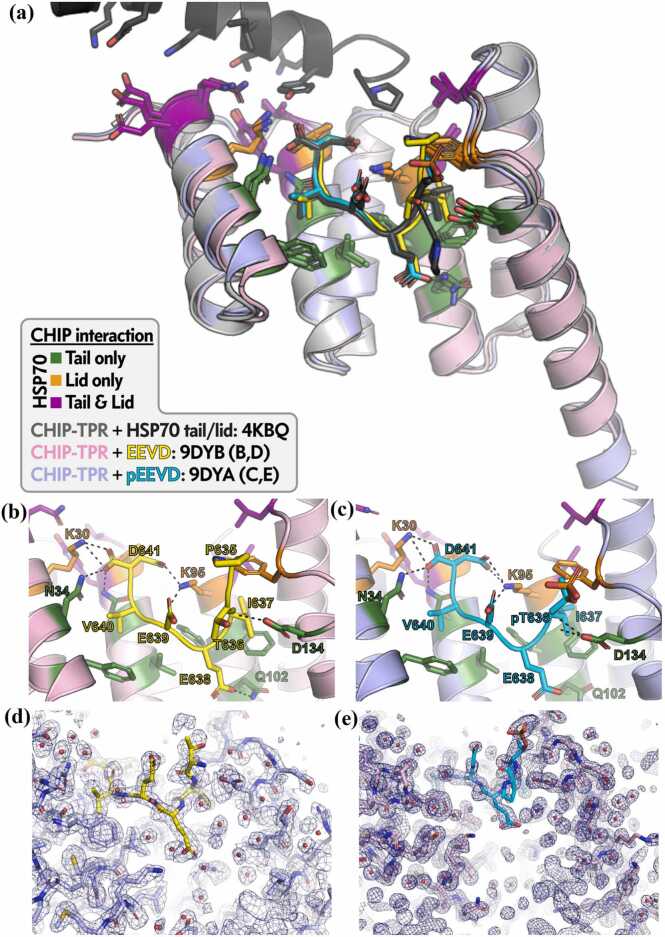
Crystal structures of CHIP-TPR bound to unphosphorylated and phosphorylated HSP70 tail peptides. (a) Overlay of CHIP-TPR structures with unphosphorylated EEVD peptide (PDB 9DYA; CHIP light purple, EEVD yellow) and phosphorylated pEEVD peptide (PDB 9DYB; CHIP light pink, pEEVD cyan). (b) Detailed interactions in the unphosphorylated complex (9DYA). (c) Detailed interactions in the phosphorylated complex (9DYB), showing disruption of key hydrogen bonds. (d) 2 F_o_-F_c_ electron density map contoured at 1σ (blue mesh) around Thr-636 in 9DYA. (e) 2 F_o_-F_c_ electron density map contoured at 1σ (blue mesh) around pThr-636 in 9DYB.

Superposition of the complexes reveals minimal backbone deviation (RMSD 0.32 Å over 128 Cα atoms) but distinct phosphate-induced rearrangements (Figure 2a). Both peptide-bound structures align closely with the prior CHIP-TPR complex containing the extended HSP70 helical lid + tail (PDB 4KBQ;^8^), confirming the canonical TPR groove interactions. In the unphosphorylated complex, the EEVD tail adopts an extended conformation, with Thr636 hydroxyl forming hydrogen bonds to CHIP Gln102 (acceptor) and Glu638 of the peptide (donor), while the peptide backbone carbonyl accepts a bond from CHIP Asp134 (Figure 2b,d).

**Fig. 3:**
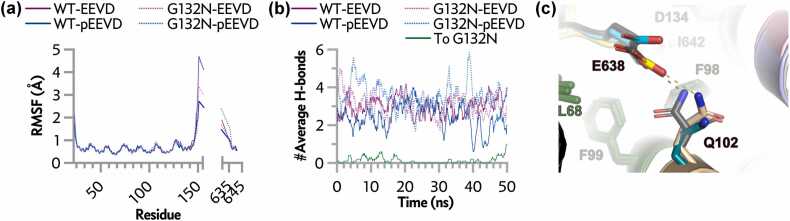
Molecular dynamics simulations of CHIP-TPR interface stability. (a) Root-mean-square fluctuation (RMSF) of Cα atoms across trajectories for complexes with unphosphorylated EEVD (purple) or phosphorylated pEEVD (blue); wild-type CHIP-TPR (solid lines), G132N variant (dotted lines). (b) Intermolecular hydrogen bond occupancy over time (ns) for the same complexes; green line shows specific contribution from N132 side chain in G132N-pEEVD complex. (c) Close-up view of differential Q102-E638 interaction: overlaid structures show hydrogen bond O-N distance of ∼2.8 Å in unphosphorylated complexes (4KBQ gray + 9DYA light blue/yellow) versus 5.8 Å in phosphorylated (9DYB light pink/cyan) due to pEEVD movement and Q102 rotation.

Phosphate addition at Thr636 abolishes both side-chain hydrogen bonds through steric hindrance and electrostatic repulsion with Asp134, forcing a ∼1.2 Å shift of the peptide away from the groove floor and rotation of Gln102 (Figure 2c, e). This rotation eliminates the Gln102-Glu638 interaction (distance increases from 2.8 to 5.8 Å; Figure 3c) and induces a minor Ile637 side-chain rotamer adjustment against the hydrophobic surface formed by CHIP Val94 and Phe98. Notably, the phosphorylated peptide conformation resembles the tail segment in PDB 4KBQ, yet Gln102 is rotated away from Glu638 here, unlike the hydrogen-bonded state in the lid-bound structure. These changes indicate inherent flexibility in Gln102 rotameric states that phosphorylation exploits to destabilize the interface. Collectively, phosphate occupancy removes two hydrogen bonds critical for high-affinity binding.

### Molecular dynamics simulations confirm interface destabilization and rescue by G132N

50 ns simulations of wild-type and G132N CHIP-TPR complexes with EEVD or pEEVD peptides showed low overall fluctuation, with expected higher mobility at termini and interhelical loops (Figure 3a). The structured TPR helices exhibited minimal deviation, while phosphorylation selectively increased Cα root-mean-square fluctuation in the tail-binding loop (residues ∼130-140).

Intermolecular hydrogen bond occupancy decreased from an average of ∼5 (unphosphorylated) to ∼2 (phosphorylated) per frame (Figure 3b). The Gln102-Glu638 interaction, persistent in unphosphorylated trajectories, was abolished upon phosphorylation (Figure 3c). In G132N-phosphorylated complexes, total hydrogen bonds increased in most frames due to direct contributions from the Asn132 side chain (Figure 3b**,** *green line*). When Asn132 engaged the phosphate, it added at least one (often two) new bonds without altering the baseline network seen in wild-type phosphorylated simulations. This confirms that affinity rescue arises specifically from phosphate coordination by the engineered asparagine. Representative trajectories illustrating interface stability and G132N rescue are provided as supplementary videos (*Videos S1-S5*).

Supplementary material related to this article can be found online at doi:10.1016/j.cstres.2026.100166.

The following is the Supplementary material related to this article Video S1, Video S2, Video S3, Video S4, Video S5..

Video S1 Supplementary material related to this article can be found online

Video S2 Supplementary material related to this article can be found online

Video S3 Supplementary material related to this article can be found online

Video S4 Supplementary material related to this article can be found online

Video S5 Supplementary material related to this article can be found online

### G132N restores binding affinity to phosphorylated peptide in isolated domains

**Fig. 4:**
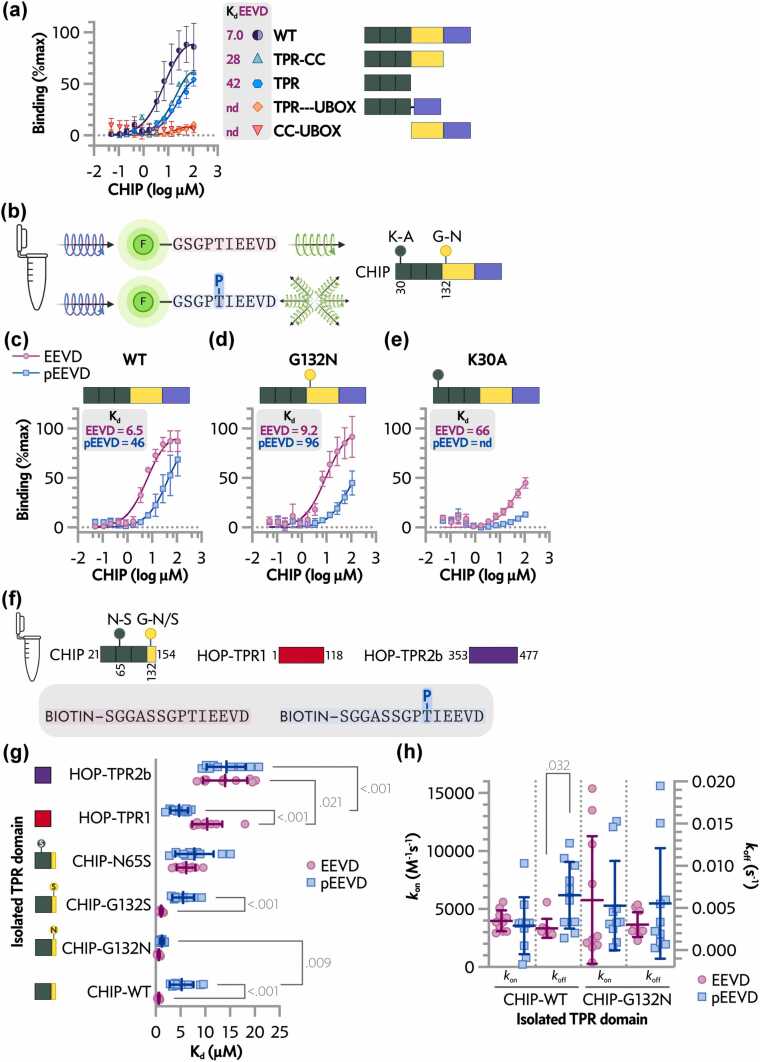
Cell-free assays reveal domain contributions to CHIP affinity and phosphorylation sensitivity restored by G132N in isolated TPR domains. (a) Dissociation constants for the unphosphorylated EEVD peptide with CHIP domain constructs. Full-length CHIP shows the highest affinity; isolated TPR shows the lowest; TPR-U-box and CC-U-box are undetectable (nd). Mean ± SD (*n* = 3). (b) Domain schematic and constructs. (c-e) Binding curves and K_d_ values for full-length wild-type (c), G132N (d), and K30A (e) CHIP with unphosphorylated EEVD or phosphorylated pEEVD peptides. Data mean ± SD (*n* = 3 independent experiments). (f) Constructs used. (g) Biolayer interferometry dissociation constants for CHIP-TPR, HOP-TPR, and G132N CHIP-TPR domains with biotinylated EEVD or pEEVD peptides. Mean ± SD (*n* ≥ 6); two-way ANOVA: TPR construct F(5, 108) = 69.54, *P* < 0.001; EEVD peptide F(1, 108) = 4.584, *P* = 0.035; interaction F(5, 108) = 10.95, *P* < 0.001. Pairwise post-test *P*-values shown on plot. (H) Biolayer interferometry dissociation k_on_ and k_off_. Mean ± SD (*n* ≥ 6); two-way ANOVA: TPR construct F(1, 36) = 0.04545, *P* = 0.832; EEVD peptide, F(1, 36) = 6.718, *P* = 0.014; interaction F(1, 36) = 0.3208. Post-test results of pairwise comparisons <0.05 are included in the plot.

Fluorescence polarization and biolayer interferometry (BLI) assays quantified the functional impact (Figure 4), with both providing equilibrium dissociation constants (K_d_) and BLI additionally enabling real-time kinetic analysis (k_off_/k_on_). Domain deletion mapping confirmed that full-length CHIP exhibits the highest affinity for unphosphorylated peptide (K_d_ 7.0 nM), with isolated TPR weakest (K_d_ 28 nM) and CC-U-box undetectable, while TPR-CC (K_d_ 42 nM) and TPR-U-box were intermediate or undetectable (Figure 4a). These data affirm bipartite tail + lid contributions and suggest that non-TPR domains (coiled-coil, U-box) can allosterically influence the interface.

In full-length CHIP, phosphorylation of the HSP70C-terminal peptide reduced affinity by 6-10 fold, and G132N did not restore binding to the phospho-peptide (pEEVD) (Figure 4b-d). The control mutation K30A abolished binding regardless of phosphorylation state (Figure 4e). Isolated wild-type CHIP-TPR bound pEEVD with more than 10-fold less affinity than the non-phosphorylated peptide (EEVD) (Figure 4f) (K_d_ 5.23 μM vs 0.592 μM; *P* = 8.7E-5). Conversely, HOP TPR domains showed varying or no sensitivity: HOP-TPR1 favored pEEVD (K_d_ 4.72 μM vs 10.4 μM; *P* = 2.0E-6), while HOP-TPR2b was insensitive (K_d_ 14.0 μM vs 14.3 μM; *P* = 0.84), aligning with known shifts toward refolding co-chaperones upon HSP70C-terminal phosphorylation. As expected, G132N CHIP-TPR recovered binding affinity to pEEVD approaching EEVD levels (K_d_ 1.25 μM vs 0.597 μM; *P* = 0.57), without affecting unphosphorylated peptide binding compared to wild-type CHIP-TPR (WT K_d_ 0.592 μM vs G132N K_d_ 0.597; *P* > 0.99) (Figure 4g). Further BLI analysis showed that rescue mainly results from increased association rates (k_on_) rather than slower dissociation (Figure 4h), indicating that rescue primarily influences association kinetics and suggests altered accessibility of the interaction interface rather than changes in complex stability. As additional controls, CHIP-G132S mimicked wild-type sensitivity, and the unrelated but TPR-disruptive mutation N65S reduced affinity equally for both peptides (Figure 4g).

Consistent with biophysical rescue in isolated domains, G132N partially mitigated the phosphomimetic-induced decrease in transient cellular interactions (NanoBiT) while overall reducing binding to the non-mimetic HSP70 but showed no rescue in steady-state co-immunoprecipitation (Figure S1). G132N retained HSP70 ubiquitination activity in vitro (Figure S2). Independently, the T636D phosphomimetic exhibited accelerated proteasome-dependent turnover relative to WT and T636A (Figure S3).

Together, these findings establish the atomic mechanism of phosphate-induced disruption at the HSP70-CHIP interface and show that structure-guided engineering fully rescues affinity only in isolated TPR domains.

## Discussion

### Atomic mechanism of phosphorylation-dependent disruption of the HSP70-CHIP interface

Our structures reveal the atomic mechanism by which phosphorylation of HSP70 Thr636 disrupts CHIP binding: phosphate-induced steric clashes and electrostatic repulsion eliminate two key hydrogen bonds (the Gln102-Thr636 hydroxyl and Gln102-Glu638, Figure 2), shifting the interface and favoring HOP recruitment (Figure 4g). This switch, induced under proteotoxic or oxidative stress, redirects client triage from degradation toward refolding, consistent with prior biochemical data.^10^, ^11^ The G132N variant restores these bonds in isolated TPR domains through direct phosphate coordination (Figure 4g, h), confirming the interface's malleability. The inability to rescue in full-length contexts (Figure 4a-e, Figure S1) indicates additional regulatory layers, including lid interactions or allosteric effects from CHIP's coiled-coil and U-box domains, which influence overall affinity.

### Study limitations

A key limitation is reliance on the T636D phosphomimetic, which reduces binding but differs in charge distribution and steric volume from authentic phosphate.^11^, ^17^, ^18^, ^19^, ^20^ Cell-free assays with phosphorylated peptides confirm the core mechanism in isolated domains, but full-length Fluorescence polarization and cellular data show no rescue by G132N as phosphorylation sensitivity persists. The full-length T636D mimetic's inherent proteasome-dependent instability (Figure S3) lowers steady-state levels, confounding binding readouts in co-immunoprecipitation and, to a lesser extent, transient NanoBiT assays (Figure S1). These constraints likely arise from bipartite lid interactions, nucleotide state (ADP enhancing affinity via lid closure),^5^, ^8^ or competition from endogenous chaperones.

### Integration with the chaperone code

These findings fit within the "chaperone code," in which multiple HSP70 PTMs (phosphorylation, acetylation, methylation, SUMOylation) crosstalk to fine-tune co-chaperone selection.^12^, ^13^, ^21^ Phosphorylation at Thr636 exemplifies this: it toggles triage during stress, with dysregulation promoting client accumulation in neurodegeneration (e.g., impaired CHIP-mediated tau degradation leading to aggregates in Alzheimer's models;^16^) or enhanced survival in cancer via refolding bias.^11^, ^14^ PTMs on CHIP and HOP further amplify this: CHIP phosphorylation modulates dimerization and client specificity,^22^, ^23^ while HOP phosphorylation enhances refolding under stress.^24^ Such interactions underscore the layered control of proteostasis, where Thr636 phosphorylation may synergize with nearby SUMOylation or acetylation to lock in co-chaperone preferences.

### Conclusions

Targeted interface engineering, as demonstrated here, provides a foundation for modulating HSP70-CHIP dynamics without global perturbation. Combining G132N with lid-targeted mutations or small-molecule stabilizers could achieve complete phospho-bypass, restoring degradative flux in stress-overloaded states. For instance, compounds mimicking G132N's phosphate coordination could selectively enhance CHIP affinity in phospho-enriched environments, bypassing the need for genetic intervention. This strategy holds promise for rebalancing proteostasis in neurodegeneration and cancer, where HSP70 hyperphosphorylation contributes to pathological triage shifts.

Our structure-guided protein-engineering method may be applicable to other chaperone/co-chaperone or chaperone/client pairs affected by disease-related PTMs. Complementary computational tools now enable the quick design of peptide-based modulators targeting these interfaces. Deep-learning pipelines have produced peptides that specifically block the oncogenic HSP70–BAG2 interaction,^25^ while de novo-designed mini-proteins can either activate or inhibit HSP70 ATPase activity to break down intracellular condensates.^26^ Structure-guided methods for modifying the HSP70 protein–protein interface further expand options for remodeling the chaperone network.^27^, ^28^ When combined with atomic-level structures such as those reported here, these techniques provide a framework for understanding and potentially modulating cellular proteostasis.

## Materials and methods

### Vectors, peptides, and recombinant proteins

Vectors are listed in Table 1. Plasmids were propagated in DH5α *Escherichia coli* and purified using Qiagen Miniprep or Bio-Rad Midiprep kits. Mutations were introduced with Q5 (NEB) or QuikChange II XL (Agilent) kits and verified by Sanger sequencing. Peptides (Lifetein/Genscript) included unphosphorylated GPTIEEVD (EEVD), phosphorylated GP(pT)IEEVD (pEEVD), biotin-GSGSGPTIEEVD (biotin-EEVD), biotin-GSGSGP(pT)IEEVD (biotin-pEEVD), and FITC-Ahx-GSGPTIEEVD or FITC-Ahx-GSGP(pT)IEEVD for polarization assays. Recombinant CHIP-TPR (residues 21-154) and full-length CHIP proteins were expressed and purified as described.^8^, ^29^

**Table 1:** Listed are the vectors used in the study.

Vector	Host	Experiment (s)	Tag	Key mutation	Lab
CHIP-TPR	Pro	Crystal structures, biolayer interferometry	HIS	Wild-type	RCP
HSP70-lid/ tail	Pro	Crystal structures, biolayer interferometry	HIS	Wild-type	RCP
CHIP	Pro	Fluorescent polarization, in vitro ubiquitination	HIS	Wild-type	JCS
CHIP-K30A	Pro	Fluorescent polarization	HIS	K30A	JCS
CHIP-G132N	Pro	Fluorescent polarization, in vitro ubiquitination	HIS	G132N	JCS
CHIP TPR-CC	Pro	Fluorescent polarization, in vitro ubiquitination	HIS	UBOX deletion	JCS
CHIP TPR-UBOX	Pro	Fluorescent polarization, in vitro ubiquitination	HIS	CC deletion	JCS
CHIP CC-UBOX	Pro	Fluorescent polarization, in vitro ubiquitination	HIS	TPR deletion	JCS
CHIP TPR	Pro	Fluorescent polarization, in vitro ubiquitination	HIS	UBOX and CC deletion	JCS
WT HSP70	Pro	In vitro ubiquitination	HIS	Wild-type	NGB
Empty Vector (pcDNA3)	Euk	Co-immunoprecipitation, cell proliferation	None	none	JCS
HSP70-WT	Euk	Co-immunoprecipitation, cell proliferation	FLAG	Wild-type	JCS
HSP70A	Euk	Co-immunoprecipitation, cell proliferation	FLAG	T636A	JCS
HSP70D	Euk	Co-immunoprecipitation, cell proliferation	FLAG	T636D	JCS
WT CHIP	Euk	Co-immunoprecipitation, cell proliferation	MYC	Wild-type	JCS
K30A CHIP	Euk	Co-immunoprecipitation, cell proliferation	MYC	K30A	JCS
G132N CHIP	Euk	Co-immunoprecipitation, cell proliferation	MYC	G132N	JCS
LgBiT-HSP70-WT	Euk	NanoBiT	LgBiT Nanoluc	Wild-type	KMS
LgBiT-HSP70-A	Euk	NanoBiT	LgBiT Nanoluc	T636A	JCS
LgBiT-WT HSP70-D	Euk	NanoBiT	LgBiT Nanoluc	T636D	JCS
SmBiT-CHIP-WT	Euk	NanoBiT	SmBiT Nanoluc	Wild-type	KMS
SmBiT-CHIP-K30A	Euk	NanoBiT	SmBiT Nanoluc	K30A	JCS
SmBiT-CHIP-G132N	Euk	NanoBiT	SmBiT Nanoluc	G132N	JCS

The experimental use, length, tag, mutation, and source of each construct are included. Vector sequences are available via Addgene. KMS, Kenneth Matt Scaglione, Duke University (matt.scaglione@duke.edu).

### X-ray crystallography

Peptides, EEVD and pEEVD, were solubilized in 20 mM HEPES, 150 mM NaCl, pH 7.0. Recombinant CHIP-TPR was mixed with peptides at a 3:1 peptide:protein ratio at a total concentration of 7 mg/ml. Co-crystallization trials were conducted using sitting drop vapor diffusion at room temperature. Crystals of CHIP-TPR/peptides grew in 0.8 μl protein/peptide mixed with 0.8 μl crystallization condition containing 0.2 M lithium sulfate, 0.1 M Tris-HCl, pH 8.5%, and 30% (w/v) PEG 4000. Crystals of CHIP-TPR/peptides tail grew in 0.8 μl protein/peptide mixed with 0.8 μl crystallization condition containing 0.2 M lithium sulfate, 0.1 M Tris-HCl, pH 8.5, 30% (w/v) PEG 4000-, and 10-mM zinc chloride.

Crystals were harvested approximately 1 week after appearance, cryoprotected in LV Cryo Oil (MiTeGen), and frozen in liquid nitrogen before data collection at the Advanced Light Source beamline 4.2.2 at Lawrence Berkeley National Laboratory. Data were processed in XDS.^30^ Structures were solved by molecular replacement using PHASER with the CHIP-TPR structure (PDB ID 4KBQ).^8^, ^31^ Structures were subjected to iterative model building and refined using COOT and PHENIX software packages.^32^, ^33^ Geometric and stereochemical validation was performed using MolProbity. Data collection and refinement statistics are provided in Table 2.

**Table 2:** X-ray data collection and structure refinement used to generate Figure 3.

	HsCHIP_21-154_ + HsHsp70_634-641_	HsCHIP_21-154_ + pT_636_–HsHsp70_634-641_
*Data collection*		
Beam line	ALS 4.2.2	ALS 4.2.2
Wavelength (Å)	1.0000	1.0000
Space group	*C*222_1_	*C*222_1_
*Cell dimensions*		
*a*, *b*, *c* (Å)	46.89, 74.36, 77.63	37.58, 45.93, 78.15
*a*, *b*, *g* (°)	90, 90, 90	90, 90, 90
Resolution (Å)^a^	39.67-1.89 (1.98-1.89)	39.60-1.59 (1.64-1.59)
*R*_*merge*_^b^	0.170 (1.014)	0.092 (0.859)
*R*_*meas*_^c^	0.198 (1.21)	0.107 (1.013)
*CC*_*1/2*_	0.988 (0.442)	0.997 (0.612)
<*I*/*sI*>	7.05 (0.99)	10.32 (1.25)
Wilson *B* factor (Å^2^)	14.66	13.58
Completeness (%)	98.93 (91.46)	99.70 (97.38)
Redundancy	3.8 (3.2)	3.7 (3.5)
No. of reflections	77,973 (7819)	127,365 (8914)
No. of unique reflections	20,723 (2406)	34,506 (2570)
*Refinement*		
No. reflections for refinement	11,040 (1242)	18,531 (1855)
*R*_*work*_/*R*_*free*_	0.189/0.234	0.173/0.213
*Average B factors*		
Protein	18.18	16.53
Water	24.47	24.95
Ions	21.11	20.47
*R.m.s deviations*		
Bond lengths (Å)	0.010	0.006
Bond angles (°)	1.160	0.780
*Ramachandran plot statistics*		
Favored regions % (#)	99.2 (131/132)	97.9 (137/140)
Allowed regions % (#)	100.0 (132/132)	100.0 (140/140)
Disallowed regions	0.0	0.0
*MolProbity validation statistics*		
Cb deviations >0.25 Å	0	0
MolProbity clash score	1.85	1.80
MolProbity clash percentile	99th percentile (*N* = 1784, all resolutions)	99th percentile (*N* = 1784, all resolutions)
MolProbity score	0.95	1.00
MolProbity score percentile	100th percentile (*N* = 27,675, all resolutions)	100th percentile (*N* = 27,675, all resolutions)
PDB ID	9DYA	9DYB

^a^Values in parentheses are for the highest resolution shell. ^b^ The merging *R* factor measures the consistency between multiple measurements of the same reflection. ^c^ The corrected *R* factor was the final adjustment used for known systematic errors in the data.

### Molecular dynamics simulations

Molecular dynamics simulations were conducted using Gromacs 2020.2.^34^ Models for CHIP-TPR/HSP70 tail, CHIP-TPR/pT-HSP70 tail, G132N-CHIP-TPR/HSP70 tail, and G132N-CHIP-TPR/pT-HSP70 tail were generated using the crystal structures solved and reported in this manuscript. The G132N mutation was generated in PyMOL by selecting the m-80° rotamer, a favored backbone-dependent rotamer.^35^ Initial coordinate files, solvation files, ions, and MD run files were generated using the CHARMM-GUI web interface.^36^ Simulations used a rectangular water box with 10.0 Å padding and sodium and chloride ions at 150 mM. A temperature of 303.15 K was used for the NVT and NPT phases during equilibration. Simulations were conducted using the CHARMM36m force field for 50 ns.^37^ Trajectories, root-mean-square fluctuation (RMSF), and hydrogen bond profiles were analyzed using VMD.^38^ Representative trajectories are provided as supplementary videos (Videos S1–S5).

### Fluorescence polarization assay

The N-terminally labeled peptides were loaded into PCR reaction tubes at 2X (40 nM), resulting in a final concentration of 20 nM. Recombinant CHIP constructs were added to the tubes at the indicated concentrations. The reaction was left at room temperature for 30 min before loading into black, low-volume, round-bottom 384-well plates (Greiner Bio-One) with inter-run triplicates at 18 μl each and subsequently repeated independently 2 more times. The Clariostar plate reader captured data using an excitation wavelength of 485 nm and an emission wavelength of 530/40 nm, with 100 flashes. The polarization readings were normalized to the dilution buffer. Gain adjustment was made to the tracer HSP70 peptides alone. We used CHIP-WT plus EEVD to calculate Bmax (289) and normalized all polarization data to % of maximum binding. The K_d_ was calculated in GraphPad Prism (v10.4.1) using a nonlinear regression for one-site total binding (B_max_ = 100; constant background = 0).

### Biolayer interferometry

Bio-layer interferometry measured peptide binding using a BLItz instrument (FortÉBio, Sartorius) with streptavidin (SA) sensors. Biotin-EEVD and biotin-pEEVD were solubilized in 20 mM HEPES, 150 mM NaCl, pH 7.0. Affinity measurements were made using the advanced kinetic protocol within the BLItz Pro system software. Biosensors were hydrated for 10 min in assay buffer comprising 20 mM HEPES, 150 mM NaCl, and 5 mM β-mercaptoethanol, pH 7.2. For each affinity measurement, a new, hydrated biosensor was placed on the BLItz tip, and the “Initial Baseline” step was completed by incubation for 30 s in 500 μl assay buffer in an opaque black microcentrifuge tube (Argos Technologies). Peptides were loaded during the “Loading Step” by incubating the tip in 4 μl of either peptide for 60 s. The “Baseline step” was then conducted by incubation for 30 s in 500 μl assay buffer in an opaque black microcentrifuge tube. The “Association step” was performed by incubating the tip in 4 μl of the appropriate TPR protein construct at 1 to 25 μM in assay buffer in an opaque black microcentrifuge tube for 120 s. The “dissociation step” was conducted by incubating the tip for 120 s in 500 μl assay buffer in an opaque black microcentrifuge tube. Affinities (K_d_) were calculated by the BLItz Pro software as K_d_ = k_off_/k_on,_ where k_off_ and k_on_ were determined by individual fits to the “dissociation step” and “association step,” respectively, for each run.

### Statistical analysis

Data were analyzed in GraphPad Prism v10.4.1 as described in the figure legends.

## Funding and support

This work was supported by the National Institutes of Health [R35GM128595 (R.C.P.), R35GM128855 (N.G.B.), R01AG066710, and R01AG061188 (J.C.S.)]; and the American Heart Association [23IPA1048749 (N.G.B.)].

## CRediT authorship contribution statement

**Mariah Stewart:** Writing – review & editing, Writing – original draft, Investigation, Formal analysis. **Chathura Paththamperuma:** Writing – review & editing, Investigation, Formal analysis. **Colleen McCann:** Investigation. **Kelsey Cottingim:** Investigation. **Huaqun Zhang:** Investigation. **Rian DelVecchio:** Investigation. **Ivy Peng:** Investigation. **Erica Fennimore:** Investigation. **Jay C. Nix:** Resources, Investigation. **Morcos N. Saeed:** Resources. **Kathleen George:** Investigation. **Katherine Makaroff:** Investigation. **Meagan Colie:** Resources. **Ethan Paulakonis:** Resources. **Michael F. Almeida:** Resources. **Adeleye J. Afolayan:** Writing – review & editing. **Nicholas G. Brown:** Writing – review & editing, Supervision. **Richard C. Page:** Writing – review & editing, Supervision, Formal analysis, Conceptualization. **Jonathan C. Schisler:** Writing – review & editing, Supervision, Formal analysis, Conceptualization.

## Declaration of generative AI and AI-assisted technologies in the writing process

During the preparation of this work, the author(s) used Grok (xAI) to edit manuscript text, suggest subsection headings, and refine figure schemes. The authors also used Illustrae.co to generate initial designs for the graphical abstract. After using these tools/services, the authors reviewed and edited the content as needed and take full responsibility for the content of the published article.

## Declarations of interest

The authors declare that they have no known competing financial interests or personal relationships that could have influenced the work reported in this paper.

## Data Availability

The atomic coordinates and structure factors for the structures reported in this study are publicly accessible in the Protein Data Bank (www.rcsb.org) under accession codes 9DYA and 9DYB. PDB9DYA | pdb_00009dya CHIP-TPR in complex with the Hsp70 tail
PDB9DYB | pdb_00009dyb CHIP-TPR in complex with the phosphorylated Hsp70 tai
